# Meeting of the Southern Section of the American Laryngological, Rhinological and Otological Society

**Published:** 1898-04

**Authors:** 


### MEETING OF THE SOUTHERN SECTION OF THE AMERICAN LARYNGOLOGICAL, RHINOLOGICAL AND OTOLOGICAL SOCIETY.

The second annual meeting of the Southern section of the above society has just been held in Atlanta, under the chairmanship of Dr. A. W. Calhoun. This society is comparatively a new organization, but even in its short career has already reached a high standard of success, as has been shown by the able contributions of its members in the special fields of Laryngology, Rhinology and Otology.

The society is distinctively national in its aims and membership, and in the latter its endeavor has been to represent the best talent from all sections of the Union. In order to foster this idea, the society has been divided into sections, representing the various parts of the country, and these meet two or three months prior to the national meeting of the whole society. There are four sections: Eastern, Middle, Western, and Southern, each having its own chairman. Last year was the first occasion of the meeting of the various sections, and the Southern section met in New Orleans under the chairmanship of Dr. W. Scheppegrell of that city, and this year it met in Atlanta.

If this last meeting of the Southern section can be an index to the success of its future meetings, then this latter has certainly been attained. The meeting was held in the ballroom of the Kimball House, and the program, which has already been printed in the previous issue of THE JOURNAL, was fully carried out with several additional papers. The papers were all practical in character, and all showed the original work of its author. The representation from the South was large, and the program showed the following States represented: Texas, Louisiana, Mississippi, Alabama, Georgia, South Carolina, Virginia, Tennessee, Kentucky, Arkansas, and New York.

This meeting developed one fact and that was the excellency of the papers from the Southern men, showing as it did that they are just as progressive in their work as our Northern *confrères*.

For some time, we think, it has been unjustly held that the Southern men are a little backward in their profession and not strictly up to date. The program of the recent meeting certainly refutes this idea, and it but exemplified the fact that future meetings in the South will rank equally in prominence as those in other portions of the country.

